# Extraction and Separation of Fucoidan from *Laminaria japonica* with Chitosan as Extractant

**DOI:** 10.1155/2013/193689

**Published:** 2013-11-24

**Authors:** Ronge Xing, Song Liu, Huahua Yu, Xiaolin Chen, Yukun Qin, Kecheng Li, Pengcheng Li

**Affiliations:** Institute of Oceanology, Chinese Academy of Sciences, Qingdao 266071, China

## Abstract

Herein the extraction method of fucoidan from *Laminaria japonica* is reported. Firstly, chitosan, chitosan-N-2-hydroxypropyl trimethyl ammonium chloride (HACC), and hexadecyltrimethylammonium bromide (CPAB) were used to extract the fucoidan. The results showed that chitosan was the optimal extractant compared with the other two extractants. After extraction, different aqueous solutions, including NaCl, KCl, and HCl (pH2), were used to separate fucoidan from chitosan-fucoidan complex. The results showed that the separation ability of NaCl was slightly higher than that of KCl. Moreover, the price of NaCl is lower than that of KCl. Given the quality-price rate, NaCl solution was chosen as the separation solution. Thirdly, the concentration and ratio of NaCl solution : sediment influence the separation of fucoidan from chitosan-fucoidan complex. The results showed that the optimal separation conditions include 4 mol/L NaCl solution with the ratio of NaCl solution to sediment at 30 : 1. Fucoidan content was found to be affected by different separation time. Fucoidan content increased with the increase of separation time, and the optimal separation time was 6 h. Compared with traditional alkali extraction method, this method not only reduces the usage of alkali and acid and alleviate environment pollution, but also has the comparable extraction yield of fucoidan. It is a potential method for extraction of fucoidan.

## 1. Introduction

The brown seaweed *Laminaria japonica Aresch*. (Laminariales) is one of the most important economic seaweeds cultured in China, and it is also widely distributed in Japan and Korea. The utilization of *L. japonica* medicine has been documented in traditional chinese medicine for over than one thousand years. In our laboratory, fucoidan was extracted from *L. japonica*. Fucoidan is a kind of sulfated fucose containing polysaccharides. Studies have shown that fucoidan has a wide spectrum of activities in biological systems including anticoagulant and antithrombotic activities, affects the inflammatory and immune systems, has antiproliferative and antiadhesive effect on cells, and protects cells from viral infection [1–8]. However, there are few reports concerning the optimization of extraction method of fucoidan from *L. japonica*. Currently, alkali extraction is the main extraction method of fucoidan in industry. A huge amount of alkali and acid were used, which severely polluted the environment. Therefore, it is important to find a better method to extract the sulfated polysaccharide from the soaked water of seaweed. In this paper, chitosan was used to extract fucoidan. Chitosan is usually obtained from waste materials, mainly shells of crabs, shrimp, and prawns from the seafood processing industry. It is an effective adsorbent due to its nontoxicity, biocompatibility, biodegradability, and its cationic nature, which enables chitosan to form a complex with anionic fucoidan. In this paper, we studied and compared the effect of different extractants, including chitosan, chitosan-N-2-hydroxypropyl trimethyl ammonium chloride (HACC), and hexadecyltrimethylammonium bromide (CPAB) on the extraction of fucoidan. Some reaction conditions and result were studied and determined as follow: the yield and content of fucoidan; the optimal dosage and flocculate time of extractant; and the separation of fucoidan from chitosan-fucoidan, HACC-fucoidan, and CPAB-fucoidan complexes. So far, no study has been reported on the extraction and separation of fucoidan from chitosan-fucoidan, HACC-fucoidan, and CPAB-fucoidan complexes.

## 2. Materials and Methods

### 2.1. Chemicals

*Laminaria japonica*, cultured in Shazikou, Qingdao, China, was collected in March, 2006, and the fresh seaweed was soon washed, dried by sun, and kept in plastic bags at room temperature for use. Chitosan from crab shells (Qingdao Yunzhou Biochem. Corp., China), which had a degree of deacetylation of 0.85 and average molecular weights of 560 KD, was used. Chitosan-N-2-hydroxypropyl trimethyl ammonium chloride (HACC) was prepared according to Xing's methods [9]. Hexadecyltrimethylammonium bromide (CPAB) was purchased from Sigma Chemical Co. HCl, NaCl, KCl, and other reagents were of analytical reagent grade and were used without further purification. All solutions were prepared with distilled water.

### 2.2. Extraction of Fucoidan

300 g dry algae were cut and soaked in 7000 mL water at rt for 24 h. After being soaked, the solution was separated by successive filtration through gauze and siliceous earth, and the clear solution was obtained. Then, 1% solution of chitosan, HACC, and CPAB was added slowly to each extract solution of 300 mL, respectively, with stirring, until no further formation of complex occurred. The mixture was placed for 2–8 h, and then the precipitates were centrifuged off, freeze-dried, and weighted. Future, 0.1 g of each sample was respectively suspended in different solution, such as different concentration and different volume NaCl or KCl or HCl (pH 2) solution, and each suspension solution was stirred for 10 h. The precipitate was centrifuged off, and the supernatant was fixed at 100 mL. In order to determine the separation effect of fucoidan from chitosan-fucoidan, HACC-fucoidan, and CPAB-fucoidan complexes by different separation solvents, the fucoidan content in the supernatant was determined.

### 2.3. Analytical Methods

#### 2.3.1. The Preparation of the Regression Equation for Fucoidan

0.01 g standard fucoidan was dissolved into 100 mL double-distilled water. Then, 0, 0.15, 0.30, 0.45, 0.60, and 0.75 mL of the solution were transferred into six test tubes, respectively. Every tube was added with double-distilled water, and the whole volume was up to 1.0 mL. Also, 4.5 mL of 87% H_2_SO_4_ aqueous solution in per test tube was added to the above mixture in ice water and shaken. One minute later, six test tubes were rapidly placed into boiling water and heated for 10 min. After they were cooled at room temperature, 0.1 mL of 3% heated cysteine chloride aqueous solution was added to the mixture, and the mixture was placed for 90 min. Their absorbance at 427 nm and 396 nm was determined, respectively (according to the appendix VA of Chinese Pharmacopeia (2000)). Then, the regression equation was made between the absorbance difference.

#### 2.3.2. The Determination of Fucoidan in Separation Solution from Separation of Chitosan-Fucoidan, HACC-Fucoidan, and CPAB-Fucoidan Complexes

1 g of dried sediments (chitosan-fucoidan, HACC-fucoidan, and CPAB-fucoidan complexes, resp.) was milled to powder and dipped in 50 mL 4 mol/L NaCl for 6 h. Then, the mixture was agitated for 2 h at room temperature and filtered. The filtrate was diluted to 100 mL, and 0.2 mL of this solution was used to determine the concentration of fucoidan (*C*, mg mL^−1^). The determined method was as in Section 2.3.1. Then, the total separation effect of fucoidan from chitosan-fucoidan, HACC-fucoidan and CPAB-fucoidan complexes in the sediment (1 g) was calculated as follows:

(1) $$\begin{array}{c} \text{the}\;\text{yield}\;\text{of}\;\text{fucoidan}\;\text{in}\;1\;\text{g}\;\text{sediment}=C\times 100. \end{array}$$

#### 2.3.3. Statistical Analysis

All determinations were carried out in triplicate. All data were expressed as means ± SD. The data were analyzed by an analysis of variance (*P* < 0.05), and the means were separated by Duncan's multiple range tests. The results were processed using Excel and STATISTICA software (statsoft Inc., 1999).

## 3. Results

### 3.1. Effect of Different Extractant on Yield and Content of Fucoidan

The fucoidan was extracted by three different extractants, including chitosan, HACC, and CPAB. Yields and fucoidan content of the extraction are shown in Table 1. Table 1 shows that flocculate activities of chitosan and HACC are more pronounced than that of CPAB. Although fucoidan content of extraction with HACC is higher than that with chitosan, the yield of extraction with chitosan is higher than that with HACC. Moreover, HACC needs a further synthesis. Therefore, given the quality-price rate, chitosan was chosen as the optimal extractant.

### 3.2. Effect of Different Ratios of Chitosan: Soaked Water of Seaweed on Yield of Fucoidan

Table 2 shows that the yield of extraction was influenced by different ratio of chitosan: soaked water of seaweed. The flocculate activity of chitosan was not related ratio of chitosan : soaked water of seaweed shown in Table 2. Chitosan has a different extraction activity with different ratios of chitosan : soaked water of seaweed. In a word, the yield of extraction increased at first and then decreased with increasing ratio of chitosan : soaked water of seaweed. At 1 : 25, the yield of extraction was the highest, about 1.68%. So, 1 : 25 is the optimal ratios of chitosan: soaked water of seaweed. This phenomenon is consistent with Napper's [10] research result. Napper's result showed that the amount of flocculant is generally bigger and better, but if the amount is too large, the surface of the colloidal particles will form steric layer due to the adsorption of excess flocculant and the occurrence of steric stabilization phenomena; therefore, this result will prevent the formation of bridging structure, and less prone to flocculation, affecting processing results. Therefore, in this paper, higher chitosan dosage is not the best.

### 3.3. Effect of Different Separation Solvents on Fucoidan Content

In this paper, different solvents were used to separate fucoidan from chitosan-fucoidan complex.  Table 3 shows that the fucoidan content changed abruptly when an inorganic salt was added to the separation solution. The results suggest that the metal ion can promote separation of chitosan-fucoidan complex. As shown in Table 3, their orders of separation on fucoidan were NaCl > KCl > HCl (pH2). NaCl solution had an optimal separation effect.

### 3.4. Effect of the Different Concentration of NaCl Solution on Fucoidan Content

Table 4 shows that the different concentration of NaCl solution affects the separation of fucoidan from chitosan-fucoidan complex. Fucoidan content increased with increasing NaCl solution concentration. From 2 mol/L to 3 mol/L, the fucoidan content increased abruptly from 1.63% to 4.7%; then, with the increasing concentration, fucoidan content slowly changed. 4 mol/L was the optimal extract concentration, which might be the effect of ionic strength. Ionic strength was stronger, and the separation effect of chitosan-fucoidan was better.

### 3.5. Effect of Different Ratios of NaCl Solution: Sediment on Fucoidan Content

Different ratios of NaCl solution : sediment also influence the separation of chitosan-fucoidan complex. In Table 5, fucoidan content increased from 10 : 1 to 40 : 1; however, fucoidan content slightly decreased from 40 : 1 to 50 : 1. Moreover, fucoidan content slightly changed from 30 : 1 to 50 : 1. Therefore, for facilitating posttreatment, 30 : 1 was the optimal ratio.

### 3.6. Effect of the Different Separation Time on Fucoidan Content

Table 6 shows the effect of the different separation time on fucoidan content. In Table 6, the fucoidan content increased with increasing separation time. From 4 h to 6 h, the fucoidan content abruptly changed; then, fucoidan content slightly changed with increasing time. Therefore, the optimal separation time is 6 h.

## 4. Discussion

Fucoidan contains substantial percentages of L-fucose and sulphate ester groups. Due to these functional groups, fucoidan has a wide spectrum of biological activities such as anticoagulant and antithrombotic activities [11], anti-inflammatory activity [12], and antitumor activity [13]. In order to obtain fucoidan, different techniques were used to extract fucoidan, which include the consumption of calcium-containing solvents, acid media, or plain water [14–16]. During the process of conventional production, NaOH was added to the soaked water of seaweed. With pH up to about 12 of the soaked water of seaweed, the polysaccharide mixture was separated [17]. This process needs abundant NaOH and produces a lot of wastewater containing NaOH. These methods not only pollute environment but also waste resources. Therefore, many researchers are seeking better methods to separate polysaccharide mixture from the soaked water. In this paper, in order to reduce the usage of NaOH, new methods to extract fucoidan and to separate it from the complex of chitosan-fucoidan were introduced. Effect of different extractants, different solvents, concentrations and volume of solution and the effect of the different separation time on yield and content of fucoidan were researched. The results indicate that chitosan is the optimal extractant, NaCl solution has better separation effect, 4 mol/L and 30 mL are the optimal concentration and volume of NaCl solution, and the optimal separation time is 6 h.

## Figures and Tables

**Table 1:** Yields and fucoidan content of extraction of different extractants.

Extractant	Yield (%)^△^	Fucoidan content (%)*
Chitosan	1.68 ± 0.02	5.51 ± 0.03
HACC	1.59 ± 0.05	5.89 ± 0.02
CPAB	0.32 ± 0.03	2.05 ± 0.03

^△^Fucoidan to dry algae. Usually the yield of fucoidan is 1-2% with the method of alkali extraction. Yield of fucoidan means extraction amount of fucoidan from the dry algae.

*12 mL extractant was used to extract fucoidan, and the extract production was suspended in 50 mL 4 mol/L NaCl. Fucoidan content means the % of fucoidan released to the aqueous solvent after breaking the complexes.

**Table 2:** Yields of extraction of different ratios of chitosan : soaked water of seaweed*.

Ratio	1 : 75	1 : 50	1 : 25	1 : 18.75	1 : 15
Yield of fucoidan (%)	0.63 ± 0.03	1.31 ± 0.05	1.68 ± 0.02	1.59 ± 0.03	0.98 ± 0.02

^∗^Concentration of chitosan is 1%.

**Table 3:** Fucoidan content of extract production in NaCl, KCl, and HCl (pH2) solution of 50 mL.

Different solvent	NaCl	KCl	HCl (pH2)
Fucoidan content (%)	5.51 ± 0.03	5.47 ± 0.02	1.03 ± 0.05

**Table 4:** Fucoidan content of separation production in the different concentration of NaCl solution (50 mL).

Concentration (mol/L)	1	2	3	4
Fucoidan content (%)	1.41 ± 0.03	1.63 ± 0.05	4.7 ± 0.02	5.51 ± 0.03

**Table 5:** Fucoidan content of separation production in different ratios of NaCl solution : sediment (4 mol/L).

Ratio	10 : 1	20 : 1	30 : 1	40 : 1	50 : 1
Fucoidan content (%)	1.91 ± 0.05	3.29 ± 0.03	5.41 ± 0.02	5.79 ± 0.03	5.51 ± 0.03

**Table 6:** Fucoidan content of extraction at a different separation time*.

Time (h)	2	4	6	8	10
Fucoidan content (%)	4.65 ± 0.01	4.75 ± 0.03	5.3 ± 0.02	5.35 ± 0.02	5.41 ± 0.03

^∗^30 mL and 4 mol/L NaCl solution were used to extract the fucoidan.

## References

[1] Usov AI, Adamyants KS, Miroshnikova LI, Shaposhnikova AA, Kochetkov NK (1971). Solvolytic desulphation of sulphated carbohydrates. *Carbohydrate Research*.

[2] Ribeiro AC, Vieira RP, Mourao PAS, Mulloy B (1994). A sulfated *α*-L-fucan from sea cucumber. *Carbohydrate Research*.

[3] Vilela-Silva ACES, Alves AP, Valente AP, Vacquier VD, Mourão PAS (1999). Structure of the sulfated *α*-L-fucan from the egg jelly coat of the sea urchin *Strongylocentrotus franciscanus*: patterns of preferential 2-O- and 4-O-sulfation determine sperm cell recognition. *Glycobiology*.

[4] Alves AP, Mulloy B, Diniz JA, Mourão PAS (1997). Sulfated polysaccharides from the egg jelly layer are species-specific inducers of acrosomal reaction in sperms of sea urchins. *The Journal of Biological Chemistry*.

[5] Lipkind GM, Shashkov AS, Nifant’ev NE, Kochetkov NK (1992). Computer-assisted analysis of the structure of regular branched polysaccharides containing 2,3-disubstituted rhamnopyranose and mannopyranose residues on the basis of ^13^C NMR data. *Carbohydrate Research*.

[6] Khatuntseva EA, Ustuzhanina NE, Zatonskii GV, Shashkov AS, Usov AI, Nifant’ev NE (2000). Synthesis, NMR and conformational studies of fucoidan fragments 1: ^1^ desulfated 2,3-and 3,4-branched trisaccharide fragments and constituting disaccharides. *Journal of Carbohydrate Chemistry*.

[7] Kovac P, Hirsch J, Shashkov AS, Usov AI, Yarotsky SV (1980). ^13^C-n.m.r. spectra of xylo-oligosaccharides and their application to the elucidation of xylan structures. *Carbohydrate Research*.

[8] Ciucanu I, Kerek F (1984). A simple and rapid method for the permethylation of carbohydrates. *Carbohydrate Research*.

[9] Xing RE, Liu S, Guo ZY (2008). Relevance of molecular weight of chitosan-*N*-2-hydroxypropyl trimethyl ammonium chloride and their antioxidant activities. *European Journal of Medicinal Chemistry*.

[10] Napper DH (1983). *Polymeric Stabilization of Colloidal Dispersions*.

[11] Chevolot L, Mulloy B, Ratiskol J, Foucault A, Colliec-Jouault S (2001). A disaccharide repeat unit is the major structure in fucoidans from two species of brown algae. *Carbohydrate Research*.

[12] Chizhov AO, Dell A, Morris HR (1999). A study of fucoidan from the brown seaweed Chorda filum. *Carbohydrate Research*.

[13] Zhuang C, Itoh H, Mizuno T, Ito H (1995). Antitumor active fucoidan from the brown seaweed, Umitoranoo (*Sargassum thunbergii*). *Bioscience, Biotechnology, and Biochemistry*.

[14] Duarte MER, Cardoso MA, Noseda MD, Cerezo AS (2001). Structural studies on fucoidans from the brown seaweed *Sargassum stenophyllum*. *Carbohydrate Research*.

[15] Zvyagintseva TN, Shevchenko NM, Popivnich IB (1999). A new procedure for the separation of water-soluble polysaccharides from brown seaweeds. *Carbohydrate Research*.

[16] Marais M, Joseleau J (2001). A fucoidan fraction from *Ascophyllum nodosum*. *Carbohydrate Research*.

[17] Xue DM, Ma JJ, Pei ZQ, Wang XL A method of cleaning the soaked water of seaweed.

